# Numerical Solution of the Problem of Relaxation Filtration of a Suspension Through a Radial Filter at a Constant Flow Velocity

**DOI:** 10.3390/membranes16070246

**Published:** 2026-07-17

**Authors:** Volodymyr Brazhenko, Bakhtiyor Kh. Khuzhayorov, Usmonali Saydullaev, Jamol Makhmudov, Iroda Beknazarova

**Affiliations:** 1 College of Engineering, Zhejiang Normal University, Jinhua 321004, China; 2 Department of Applied Mathematics, Faculty of Artificial Intelligence and Digital Technologies, Samarkand State University named after Sharof Rashidov, 15 University Street, Samarkand 140100, Uzbekistanusmonali.jurayevich@gmail.com (U.S.);; 3 V.I. Romanovskiy Institute of Mathematics, Uzbekistan Academy of Sciences, Tashkent 100174, Uzbekistan; 4 Faculty of English, Samarkand State Institute of Foreign Languages, 43 Gagarin Street, Samarkand 140104, Uzbekistan; irodabeknazarova77@gmail.com

**Keywords:** relaxation filtration, constant flow velocity, relaxation cake, moving boundary problem, Darcy relaxation law

## Abstract

This paper investigates the relaxation filtration of a suspension through a radial filter surface under conditions of constant flow velocity. A mathematical model for relaxation cake growth is formulated based on the liquid-phase continuity equation, Darcy’s relaxation law, and constitutive relations for both compressive and liquid pressures. The resulting governing equation is a nonlinear partial differential equation for the compressive pressure, complemented by a Stefan condition that characterizes the motion of the cake–slurry interface. The moving-boundary problem is solved numerically using a finite difference method employing a coordinate-based front-tracking technique combined with iterative procedures. The numerical results demonstrate the influence of relaxation effects on cake formation. Increasing the relaxation time slows the compaction process, thereby maintaining higher porosity and promoting accelerated growth of the cake layer thickness.

## 1. Introduction

Filtration is the process of separating solid particles from liquids by passing a suspension through a permeable medium that retains the particles. Particles retained on the surface of the medium form a cake layer. During filtration, the cake thickness increases [1]. If the resulting cake is compressible, its structure, permeability, and solids content also change over time under the influence of pressure. At a fixed filtration pressure, the suspension flow rate decreases over time, and to ensure a constant flow rate, a corresponding gradual increase in the applied pressure is required [2,3].

The mathematical model of sediment formation within the framework of classical filtration theory is represented by a set of the following equations [1,4,5,6,7,8,9]: the material balance equations for the liquid and solid phases, the force balance equation, and the Darcy equation, and also includes the dependence of the solid content and permeability on the compression pressure [10,11,12]. In the works of a number of scientists [13,14,15,16,17,18,19], models have been proposed that take into account the complex physical and mechanical nature of the interaction of phases in the sediment.

A simplified model of filtration with sediment formation is presented in [6], which takes into account the effect of pressure redistribution at the sediment-filter medium boundary. The obtained analytical solutions allow for a more accurate prediction of the sediment growth process compared to classical theory. An analytical review of the literature on filtration process modeling is presented in [7], which provides a comparative analysis of traditional and modern approaches to deriving fundamental equations of mass and momentum balance, taking into account the functional dependencies of the sediment layer parameters. A critical analysis of the traditional theory of filtration with sediment formation is presented in the work of Teoh et al. [8].

A number of works by R. Burger and co-authors [20,21,22] are devoted to the development of a unified phenomenological theory combining the processes of sedimentation and filtration. In works [23], a development of the filtration theory was proposed that for the first time included consideration of irreversible plastic deformations of the sediment layer along with its elastic properties. The sediment was considered as an elastic-plastic medium, for which equations were derived that describe the processes of primary loading (sediment formation), unloading, and reloading. By solving an axisymmetric problem, the authors were able to evaluate in detail and quantitatively the influence of the plastic properties of the cake layer on the rate of growth of its thickness and the kinetic characteristics of filtration. In works [24], equations for the filtration of suspensions with the formation of a relaxing sediment were derived based on conservation laws. The Stefan problem was posed to describe the dynamics of sediment thickness growth. The influence of relaxation time on cake layer parameters, such as the distribution of pressure, porosity, and permeability across the sediment thickness, was assessed. A mathematical model based on plasticity theory was proposed in [25] for analyzing transient distributions of stress, strain, and porosity in a sediment layer under constant axial loads.

In addition, the present study is relevant to dynamic filtration systems, where controlled hydrodynamics are used to regulate cake formation. Previous studies [26,27] have demonstrated that rotation and suction in porous cylindrical configurations significantly modify the flow structure and transport processes. This suggests that incorporating relaxation effects into filtration models may be essential for describing cake evolution under such conditions.

This paper examines the relaxation filtration of a suspension through a radial filter at a constant flow rate. A relaxation cake layer forms on the filter surface, the growth of which is described by the function $R\left(t\right)$. The process is one-dimensional and radially symmetric, with the liquid phase moving in the direction of decreasing radial coordinates. Based on the numerical solution of the relaxation filtration equations, the distributions of the compressive pressure across the cake thickness, as well as the solid phase concentration and permeability within the layer, are obtained. The results demonstrate the significant influence of relaxation effects on the characteristics of cake layer formation.

## 2. Filtration Equations with the Formation of a Relaxation Cake Layer

**Figure 1:**
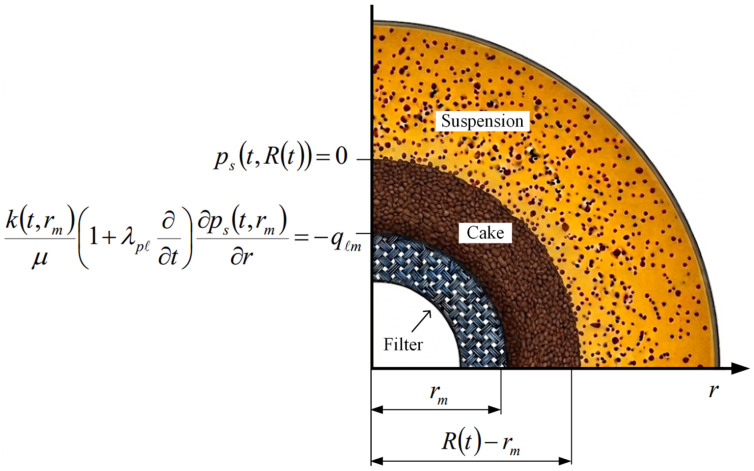
Schematic of the filtration process with the presented boundary conditions.

Consider the process of filtration (Figure 1) of a suspension through a cylindrical surface of radius $r_{m}$. Filtration occurs in the radial direction toward the center (in the direction of decreasing radius *r*). Suspension particles are retained on the outer surface of the filter, forming a cylindrical cake layer, which occupies the region $r_{m}\leq r\leq R\left(t\right)$, where $R\left(t\right)$ is the boundary between the sediment and the suspension. The thickness of the sediment layer continuously increases with time. The suspension is located in the region $r>R\left(t\right)$. The flow is considered one-dimensional and radially axially symmetric. Since the liquid moves toward the center of the cylinder, the velocity vectors of the liquid phase $q_{l}$ and solid particles $q_{s}$ are negative relative to the axis r.

To describe fluid flow in porous media, filtration theory relies on various models of continuous media, subjecting their motion to strictly defined physical laws. This allows for the transition from studying the motion of individual particles in channels to a macroscopic description of flow within a reservoir. The most commonly used model of a continuous medium is the Newtonian fluid. Its motion in a porous medium obeys the classical linear law of filtration—Darcy’s law [1,6,8,19]. In general, this relationship is expressed by the following equation:

(1) $$\frac{q_{ls}}{1-\varepsilon_{s}}=\frac{q_{l}}{1-\varepsilon_{s}}-\frac{q_{s}}{\varepsilon_{s}}=\frac{1}{1-\varepsilon_{s}}\frac{k}{\mu }\frac{\partial p_{l}}{\partial r},$$

where $q_{ls}$ is the total filtration rate, $q_{l}$ is the filtration rate of the liquid phase, $q_{s}$ is the filtration rate of the solid phase, $\varepsilon_{s}$ is the relative content of the solid phase, k is the permeability, μ is the viscosity of the suspension, $p_{l}$ is the pressure in the liquid phase, r is the radial coordinate.

Equation of motion (1) is based on the principles of direct proportionality between filtration rate and pressure gradient, as well as on the hypothesis of instantaneous establishment of equilibrium between $q_{ls}$ and ∂pl∂r. This means that with any change in one of the parameters, the system instantly, without a time lag, transitions to a new state. Obviously, Equation (1) provides a description of filtration flows in homogeneous media only in cases where the boundary and other conditions of motion remain stationary or change sufficiently slowly over time. Violation of these conditions can lead to significant errors in the hydrodynamic description of the process under study when using a linear law [28,29,30].

Although Darcy’s law assumes instantaneous adaptation of the system, in practice, equilibrium is achieved with a certain delay. This is due to a number of factors, among which pressure relaxation plays a key role, manifested as a time lag between the change in pressure gradient and the corresponding filtration rate. Identifying and correctly accounting for these effects are of fundamental importance in studying the filtration of non-Newtonian media [30,31,32,33]. The flow of a suspension in a cake layer can be described within the framework of a continuous medium model-a Maxwellian fluid, the motion of which in the pore space obeys a modified filtration law that takes into account relaxation processes [23,24]:

(2) $$\frac{q_{l}}{1-\varepsilon_{s}}-\frac{q_{s}}{\varepsilon_{s}}=-\frac{1}{1-\varepsilon_{s}}\frac{k}{\mu }\left(1+\lambda_{pl}\frac{\partial }{\partial t}\right)\frac{\partial p_{l}}{\partial r},$$

where $\lambda_{pl}$ is the relaxation time of the pressure gradient, t is the time.

Equation (2) describes the filtration process in a manner analogous to the Maxwell fluid model, where the parameter $\lambda_{p}$ denotes the pressure relaxation time. The physical meaning of this parameter is as follows: when the filtration flow at a given point is suddenly stopped, the pressure gradient does not vanish instantaneously but rather decays gradually over time according to the following law: $\frac{\partial p}{\partial r}\approx a\exp\left(-\frac{t}{\lambda_{p}}\right)$. Taking delay (lag) effects into account, the boundary conditions for pressure in the unsteady filtration process are determined based on relation (2). The pressure relaxation time depends on a number of factors, including the size of the molecules constituting the medium, the nature of the external forces acting on it, the type of porous medium, the geometric characteristics of the pore structure, and other similar factors [33].

The Darcy Equation (2) can be rewritten as

(3) $$q_{l}=\frac{1-\varepsilon_{s}}{\varepsilon_{s}}q_{s}-\frac{k}{\mu }\left(1+\lambda_{pl}\frac{\partial }{\partial t}\right)\frac{\partial p_{l}}{\partial r},$$

From the problem statement it follows that $q_{l}+q_{s}=q_{lm}=const$ (the condition of constant flow velocity). Taking this relationship into account, Equation (3) takes the form:

(4) $$q_{l}=-\varepsilon_{s}\frac{k}{\mu }\left(1+\lambda_{pl}\frac{\partial }{\partial t}\right)\frac{\partial p_{l}}{\partial r}+q_{lm}\left(1-\varepsilon_{s}\right).$$

The filtration equation is based on the continuity equations for the liquid phase, written as follows:

(5) $$\frac{\partial \varepsilon_{s}}{\partial t}=\frac{1}{r}\frac{\partial }{\partial r}\left(rq_{l}\right),$$

Substituting (4) into (5) we obtain

(6) $$\frac{\partial \varepsilon_{s}}{\partial t}=\frac{1}{r}\frac{\partial }{\partial r}\left(-r\left(\varepsilon_{s}\frac{k}{\mu }\left(1+\lambda_{pl}\frac{\partial }{\partial t}\right)\frac{\partial p_{l}}{\partial r}+q_{lm}\left(1-\varepsilon_{s}\right)\right)\right)$$

or

(7) $$-\frac{1}{r}\frac{\partial }{\partial r}\left(r\varepsilon_{s}\frac{k}{\mu }\left(1+\lambda_{pl}\frac{\partial }{\partial t}\right)\frac{\partial p_{l}}{\partial r}\right)+q_{lm}\frac{1}{r}\frac{\partial }{\partial r}\left(r\left(1-\varepsilon_{s}\right)\right)=\frac{\partial \varepsilon_{s}}{\partial t},$$

This is the basic equation of filtration in radial coordinates, describing the process of formation of a relaxation cake layer.

## 3. Derivation of the Condition for a Moving Boundary R(t)

At the cake layer/suspension interface ($r=R\left(t\right)$), the solids content changes abruptly from the value in the suspension $\varepsilon_{s_{0}}$ to the value in the sediment $\varepsilon_{s}^{0}$. This condition determines the time dependence of cake layer growth and is formulated based on the law of conservation of mass at the interface between the suspension and the cake layer [1]:

(8) $$\frac{dR}{dt}=\frac{q_{l_{i}}-q_{l_{o}}}{\varepsilon_{s_{0}}-\varepsilon_{s}^{0}},$$

where $q_{l_{i}}$ and $q_{l_{o}}$ are the filtration rates of the liquid phase in the cake layer and suspension, respectively.

Darcy’s law is used to describe the filtration of a suspension in a cake layer [24]

(9) $$q_{l_{i}}-\frac{1-\varepsilon_{s}^{0}}{\varepsilon_{s}^{0}}q_{s_{i}}=-{\left(\frac{k}{\mu }\left(1+\lambda_{pl}\frac{\partial }{\partial t}\right)\frac{\partial p_{l}}{\partial r}\right)}_{r<R},$$

where $q_{s_{i}}$ is the filtration rate of the solid phase into the cake layer.

We use the condition of constant flow velocity, i.e., $q_{l_{i}}+q_{s_{i}}=q_{lm}$, from which we have $q_{s_{i}}=q_{lm}-q_{l_{i}}$.

Taking this into account, from (9) we obtain

$$q_{l_{i}}-\frac{1-\varepsilon_{s}^{0}}{\varepsilon_{s}^{0}}\left(q_{lm}-q_{l_{i}}\right)=-{\left(\frac{k}{\mu }\left(1+\lambda_{pl}\frac{\partial }{\partial t}\right)\frac{\partial p_{l}}{\partial r}\right)}_{r<R},$$

from which it follows that

(10) $$q_{l_{i}}=-{\left(\varepsilon_{s}^{0}\frac{k}{\mu }\left(1+\lambda_{pl}\frac{\partial }{\partial t}\right)\frac{\partial p_{l}}{\partial r}\right)}_{r<R}+\left(1-\varepsilon_{s}^{0}\right)q_{lm}.$$

In the suspension zone, we assume that the velocities of the liquid and solid components are proportional to their volume fractions:

$$\frac{q_{l_{o}}}{q_{s_{o}}}=\frac{1-\varepsilon_{s}^{0}}{\varepsilon_{s}^{0}},$$

where $q_{s_{o}}$ is the filtration rate of the solid phase in the suspension.

Since $q_{l_{o}}+q_{s_{o}}=q_{lm}$, then

(11) $$q_{l_{o}}=\left(1-\varepsilon_{s_{o}}\right)q_{lm}.$$

Substituting (10) and (11) into (8), we obtain:

$$\frac{dR}{dt}=\frac{-{\left(\varepsilon_{s}^{0}\frac{k}{\mu }\left(1+\lambda_{pl}\frac{\partial }{\partial t}\right)\frac{\partial p_{l}}{\partial r}\right)}_{r<R}+\left(1-\varepsilon_{s}^{0}\right)q_{lm}-\left(1-\varepsilon_{s_{o}}\right)q_{lm}}{\varepsilon_{s_{0}}-\varepsilon_{s}^{0}}.$$

The equation for the growth of the relaxation cake layer thickness is obtained in the form:

(12) $$\frac{dR}{dt}=-\frac{\varepsilon_{s}^{0}}{\varepsilon_{s_{0}}-\varepsilon_{s}^{0}}{\left[\frac{k}{\mu }\left(1+\lambda_{pl}\frac{\partial }{\partial t}\right)\frac{\partial p_{l}}{\partial r}\right]}_{r<R}+q_{lm}.$$

The initial and boundary conditions for solving the system of Equations (7) and (12) are as follows. At the initial moment of the filtration process, the suspension does not penetrate the filter membrane, so the cake layer thickness is zero, and the solid-phase pressure is zero:

(13) $$R\left(t\right)=r_{m},p_{s}\left(0,r_{m}\right)=0.$$

At the cake-suspension boundary and on the filter partition, the following conditions are met:

(14) $$\varepsilon_{s}\left(t,R\left(t\right)\right)=\varepsilon_{s}^{0},p_{s}\left(t,R\left(t\right)\right)=0,\frac{k\left(t,r_{m}\right)}{\mu }\left(1+\lambda_{pl}\frac{\partial }{\partial t}\right)\frac{\partial p_{s}\left(t,r_{m}\right)}{\partial r}=-\;q_{lm}.$$

In the momentum conservation equations for the phases, we neglect inertial effects, viscous friction forces, and the action of mass forces. Considering that the interphase interaction forces are mutually opposite, after summing the equations for both phases, we obtain a zero sum of the acting forces and the following relationship [1]:

(15) $$dp_{l}+dp_{s}=0.$$

Cake layer parameters such as solids content and permeability depend on the compressive pressure, i.e., [1]:

(16) $$\varepsilon_{s}=\varepsilon_{s}^{0}{\left(1+\frac{p_{s}}{p_{A}}\right)}^{\beta },k=k^{0}{\left(1+\frac{p_{s}}{p_{A}}\right)}^{-\delta },$$

where $\varepsilon_{s}^{0}$, $k^{0}$ are the values of $\varepsilon_{s}$, k at $p_{s}=0$, respectively, $p_{A}$ is the characteristic pressure, β and δ are the indicators–constant values.

Taking into account relations (15) and (16), Equations (7) and (12) for the moving boundary, as well as the initial (13) and boundary (14) conditions, are reduced to the following form:

(17) $$\frac{\partial p_{s}}{\partial t}=\frac{p_{A}k^{0}}{\mu \cdot \beta }{\left(1+\frac{p_{s}}{p_{A}}\right)}^{1-\beta }\frac{1}{r}\frac{\partial }{\partial r}\left(r{\left(1+\frac{p_{s}}{p_{A}}\right)}^{\beta -\delta }\left(1+\lambda_{pl}\frac{\partial }{\partial t}\right)\frac{\partial p_{s}}{\partial r}\right)-\;-q_{lm}\frac{\partial p_{s}}{\partial r}+\frac{p_{A}}{\varepsilon_{s}^{0}\beta }\frac{q_{lm}}{r}\left({\left(1+\frac{p_{s}}{p_{A}}\right)}^{1-\beta }-\varepsilon_{s}^{0}\left(1+\frac{p_{s}}{p_{A}}\right)\right),$$

(18) $$\frac{dR}{dt}=\frac{\varepsilon_{s}^{0}}{\varepsilon_{s}^{0}-\varepsilon_{s_{0}}}{\left[\frac{k^{0}}{\mu }{\left(1+\frac{p_{s}}{p_{A}}\right)}^{-\delta }\left(1+\lambda_{pl}\frac{\partial }{\partial t}\right)\frac{\partial p_{s}}{\partial r}\right]}_{r<R}+q_{lm},$$

(19) $$p_{s}\left(0,r_{m}\right)=0,p_{s}\left(t,R\left(t\right)\right)=0,\frac{k^{0}}{\mu }{\left(1+\frac{p_{s}\left(t,r_{m}\right)}{p_{A}}\right)}^{-\delta }\left(1+\lambda_{pl}\frac{\partial }{\partial t}\right)\frac{\partial p_{s}\left(t,r_{m}\right)}{\partial r}=-\;q_{lm}.$$

We introduce the following notations

$$a\left(p_{s}\right)=\frac{p_{A}k^{0}}{\beta \mu }{\left(1+\frac{p_{s}}{p_{A}}\right)}^{1-\beta },c\left(p_{s}\right)=\frac{p_{A}}{\varepsilon_{s}^{0}\beta }\left({\left(1+\frac{p_{s}}{p_{A}}\right)}^{1-\beta }-\varepsilon_{s}^{0}\left(1+\frac{p_{s}}{p_{A}}\right)\right),$$

$$b\left(p_{s}\right)={\left(1+\frac{p_{s}}{p_{A}}\right)}^{\beta -\delta },d\left(p_{s}\right)=\frac{\varepsilon_{s}^{0}}{\varepsilon_{s}^{0}-\varepsilon_{s_{0}}}\frac{k^{0}}{\mu }{\left(1+\frac{p_{s}}{p_{A}}\right)}^{-\delta },g\left(p_{s}\right)=\frac{k^{0}}{\mu }{\left(1+\frac{p_{s}\left(t,r_{m}\right)}{p_{A}}\right)}^{-\delta }.$$

Taking these notations into account, Equations (17)–(19) are transformed to the following form

(20) $$\frac{\partial p_{s}}{\partial t}=a\left(p_{s}\right)\frac{1}{r}\frac{\partial }{\partial r}\left(b\left(p_{s}\right)r\left(1+\lambda_{pl}\frac{\partial }{\partial t}\right)\frac{\partial p_{s}}{\partial r}\right)-q_{lm}\frac{\partial p_{s}}{\partial r}+\frac{q_{lm}}{r}c\left(p_{s}\right),\;\frac{dR}{dt}={\left[d\left(p_{s}\right)\left(1+\lambda_{pl}\frac{\partial }{\partial t}\right)\frac{\partial p_{s}}{\partial r}\right]}_{r=R^{-}}+q_{lm},\;p_{s}\left(0,r_{m}\right)=0,p_{s}\left(t,R\left(t\right)\right)=0,g\left(p_{s}\right)\left(1+\lambda_{pl}\frac{\partial }{\partial t}\right)\frac{\partial p_{s}\left(t,r_{m}\right)}{\partial r}=-\;q_{lm}.$$

The solution of the system of Equation (20) determines the main characteristics of the filtration process.

**Figure 2:**
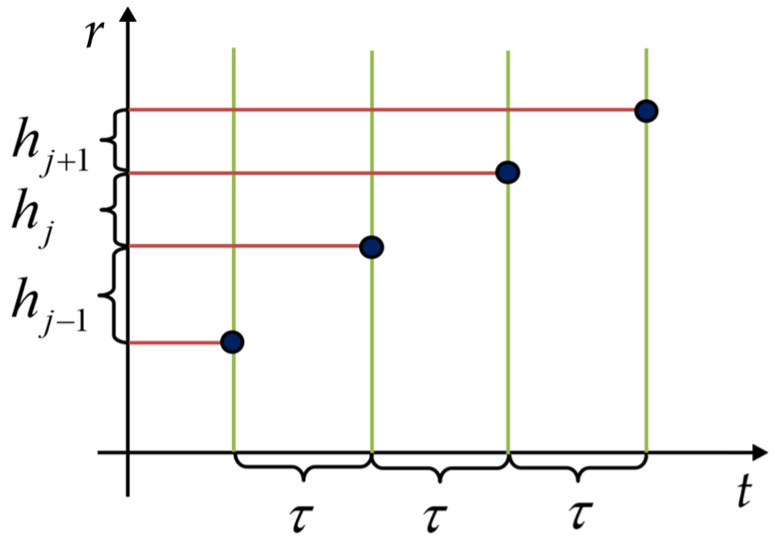
Finite-difference stencil on a grid: uniform in t and non-uniform in r.

To solve problem (20), we use the finite difference method [23,24]. The presence of an unknown moving boundary in the problem necessitates the use of a modification of the method with “front catching” along the spatial coordinate r. According to the algorithm, new nodes of the computational grid are selected such that the position of the moving boundary coincides with one of the nodes (Figure 2). Suppose that at the time moment $t=t_{j+1}$ the grid contains nodes $x_{i}=x_{i-1}+h_{i},\;i=1,2,\ldots ,i_{j+1}$, while the step $h_{n}$ for the boundary node $x_{n}$, $n=i_{j+1}$ is not determined in advance.

We introduce the notation $\theta_{i}^{j}$ for the grid function corresponding to the pressure $p_{s}$ at the point $\left(t_{j},x_{i}\right)$. Equation (20) is approximated using an implicit difference scheme that is nonlinear with respect to the desired function $\theta_{i}^{j+1}$.

(21) $$\frac{\theta_{i}^{j+1}-\theta_{i}^{j}}{\tau_{j+1}}=\frac{2a\left(\theta_{i}^{j+1}\right)}{r_{i}\left(h_{i}+h_{i+1}\right)}\left\{b(\theta_{i+1/2}^{j+1})r_{i+1/2}\left(\frac{\theta_{i+1}^{j+1}-\theta_{i}^{j+1}}{h_{i+1}}+\frac{\lambda_{pl}}{\tau }\left(\frac{\theta_{i+1}^{j+1}-\theta_{i}^{j+1}}{h_{i+1}}-\frac{\theta_{i+1}^{j}-\theta_{i}^{j}}{h_{i+1}}\right)\right)-\right.\;\left.-b(\theta_{i-1/2}^{j+1})r_{i-1/2}\left(\frac{\theta_{i}^{j+1}-\theta_{i-1}^{j+1}}{h_{i}}+\frac{\lambda_{pl}}{\tau }\left(\frac{\theta_{i}^{j+1}-\theta_{i-1}^{j+1}}{h_{i}}-\frac{\theta_{i}^{j}-\theta_{i-1}^{j}}{h_{i}}\right)\right)\right\}-q_{lm}\;\frac{\theta_{i}^{j+1}-\theta_{i-1}^{j+1}}{h_{i}}+\;+q_{lm}\frac{c\left(\theta_{i}^{j+1}\right)}{r_{i}}=0,1<i<n,\;\frac{h_{n}}{\tau_{j+1}}+d\left(\theta_{i-1/2}^{j+1}\right)\left(\frac{\theta_{n}^{j+1}-\theta_{n-1}^{j+1}}{h_{n}}+\frac{\lambda_{pl}}{\tau }\left(\frac{\theta_{n}^{j+1}-\theta_{n-1}^{j+1}}{h_{n}}-\frac{\theta_{n}^{j}-\theta_{n-1}^{j}}{h_{n}}\right)\right)+q_{lm}=0,\;\theta_{i}^{0}=0,i=0,1,\ldots ,n,\;\theta_{i}^{j+1}=0,i=n+1,\;n+2,\ldots ,j=0,1,2,\ldots ,\;g\left(\theta_{0}^{j}\right)\left(\frac{\theta_{1}^{j+1}-\theta_{0}^{j+1}}{h_{1}}+\frac{\lambda_{pl}}{\tau }\left(\frac{\theta_{1}^{j+1}-\theta_{0}^{j+1}}{h_{1}}-\frac{\theta_{1}^{j}-\theta_{0}^{j}}{h_{1}}\right)\right)=-q_{lm},j=\bar{0,N},$$

where

$$r_{i-1/2}=\frac{r_{i-1}+r_{i}}{2},r_{i+1/2}=\frac{r_{i+1}+r_{i}}{2},b\left(\theta_{i+1/2}^{j+1}\right)=\frac{1}{2}\left[{\left(1+\frac{\theta_{i+1}^{j+1}}{p_{A}}\right)}^{\beta -\delta }+{\left(1+\frac{\theta_{i}^{j+1}}{p_{A}}\right)}^{\beta -\delta }\right],\;a\left(\theta_{i}^{j+1}\right)=\frac{p_{A}k^{0}}{\beta \mu }{\left(1+\frac{\theta_{i}^{j+1}}{p_{A}}\right)}^{1-\beta },c\left(\theta_{i}^{j+1}\right)=\frac{p_{A}}{\varepsilon_{s}^{0}\beta }\left({\left(1+\frac{\theta_{i}^{j+1}}{p_{A}}\right)}^{1-\beta }-\varepsilon_{s}^{0}\left(1+\frac{\theta_{i}^{j+1}}{p_{A}}\right)\right),\;d\left(\theta_{i-1/2}^{j}\right)=\frac{\varepsilon_{s}^{0}}{\varepsilon_{s}^{0}-\varepsilon_{s_{0}}}\frac{k^{0}}{2\mu }\left[{\left(1+\frac{\theta_{i}^{j}}{p_{A}}\right)}^{-\delta }+{\left(1+\frac{\theta_{i-1}^{j}}{p_{A}}\right)}^{-\delta }\right].$$

To simplify the numerical implementation and transition to a deterministic grid, a change in variable $\frac{dR}{dt}\approx \frac{h_{n}}{\tau_{j+1}}$ is introduced.

The resulting system of equations is nonlinear, so a simple iteration method is used to solve it. Accordingly, system (21) can be rewritten in the following form:

(22) $$\frac{\theta_{i}^{\left(\sigma +1\right),j+1}-\theta_{i}^{j}}{\tau_{j+1}}-\frac{2a\left(\theta_{i}^{j+1}\right)}{r_{i}\left(h_{i}+h_{i+1}\right)}\left\{b\left(\theta_{i+1/2}^{\left(\sigma \right),j+1}\right)r_{i+1/2}\left(\frac{\theta_{i+1}^{\left(\sigma +1\right),j+1}-\theta_{i}^{\left(\sigma +1\right),j+1}}{h_{i+1}}\right.\right.+\frac{\lambda_{pl}}{\tau }\times \;\times \left.\left(\frac{\theta_{i+1}^{\left(\sigma +1\right),j+1}-\theta_{i}^{\left(\sigma +1\right),j+1}}{h_{i+1}}-\frac{\theta_{i+1}^{\left(\sigma s+1\right),j}-\theta_{i}^{\left(\sigma +1\right),j}}{h_{i+1}}\right)\right)-b\left(\theta_{i-1/2}^{\left(\sigma \right),j+1}\right)r_{i-1/2}\left(\frac{\theta_{i}^{\left(\sigma +1\right),j+1}-\theta_{i-1}^{\left(\sigma +1\right),j+1}}{h_{i}}\right.+\;\left.+\frac{\lambda_{pl}}{\tau }\left.\left(\frac{\theta_{i}^{\left(\sigma +1\right),j+1}-\theta_{i-1}^{\left(\sigma +1\right),j+1}}{h_{i}}-\frac{\theta_{i}^{\left(\sigma +1\right),j}-\theta_{i-1}^{\left(\sigma +1\right),j}}{h_{i}}\right)\right)\right\}+q_{lm}\;\frac{\theta_{i+1}^{\left(\sigma +1\right),j+1}-\theta_{i-1}^{\left(\sigma +1\right),j+1}}{2h_{i}}-\;-q_{lm}\frac{c\left(\theta_{i}^{\left(\sigma \right),j+1}\right)}{r_{i}}=0,1<i<n-1,$$

where

$$b\left(\theta_{i+1/2}^{\left(\sigma \right),j+1}\right)=\frac{1}{2}\left[{\left(1+\frac{\theta_{i+1}^{\left(\sigma \right),j+1}}{p_{A}}\right)}^{\beta -\delta }+{\left(1+\frac{\theta_{i}^{\left(\sigma \right),j+1}}{p_{A}}\right)}^{\beta -\delta }\right],\;c\left(\theta_{i}^{\left(\sigma \right),j+1}\right)=\frac{p_{A}}{\varepsilon_{s}^{0}\beta }\left({\left(1+\frac{\theta_{i}^{\left(\sigma \right),j+1}}{p_{A}}\right)}^{1-\beta }-\varepsilon_{s}^{0}\left(1+\frac{\theta_{i}^{\left(\sigma \right),j+1}}{p_{A}}\right)\right),$$

σ is iteration number.

Note that system (22) is linear with respect to the quantities $\theta_{i+1}^{\left(\sigma \right),j+1}$, which allows the sweep method to be used. The following condition can be used as a criterion for stopping the iterations at a given time layer:

(23) $$\underset{i}{max}\left|\theta_{i}^{\left(\sigma +1\right),j+1}-\theta_{i}^{\left(\sigma \right),j+1}\right|\leq \Delta ,$$

where Δ is the specified calculation accuracy. If condition (23) is satisfied, it can be assumed that $\theta_{i+1}^{\left(\sigma \right),j+1}\approx \theta_{i}^{j+1}$. The initial approximation is taken to be $\theta_{i}^{\left(0\right),j+1}=\theta_{i}^{j}$. Using the notation $y_{i}=\theta_{i}^{\left(\sigma \right),j+1}$, the difference scheme (22) is reduced to a tridiagonal system of linear algebraic equations:

(24) $$\begin{array}{c} C_{0}y_{0}-B_{0}y_{1}=F_{0},\\ -A_{i}y_{i-1}+C_{i}y_{i}-B_{i}y_{i+1}=F_{i},\;\;i=1,2,\ldots ,n-1,\\ -A_{n}y_{n-1}+C_{n}y_{n}=F_{n}, \end{array}$$

where

$$A_{i}=\frac{a\left(\theta_{i}^{j+1}\right)}{r_{i}}\frac{2}{h_{i}+h_{i+1}}\frac{b\left(\theta_{i-1/2}^{\left(\sigma \right),j+1}\right)}{h_{i}}r_{i-1/2}\left(1+\frac{\lambda_{pl}}{\tau }\right)+\frac{1}{2h_{i}}q_{lm},\;B_{i}=\frac{a\left(\theta_{i}^{j+1}\right)}{r_{i}}\frac{2}{h_{i}+h_{i+1}}\frac{b\left(\theta_{i+1/2}^{\sigma ,j+1}\right)}{h_{i+1}}r_{i+1/2}\left(1+\frac{\lambda_{pl}}{\tau }\right)-\frac{1}{2h_{i}}q_{lm},\;C_{i}=\frac{1}{\tau_{j+1}}+\frac{a\left(\theta_{i}^{j+1}\right)}{r_{i}}\frac{2}{h_{i}+h_{i+1}}\left(1+\frac{\lambda_{pl}}{\tau }\right)\left[\frac{b\left(\theta_{i+1/2}^{\left(\sigma \right),j+1}\right)}{h_{i+1}}r_{i+1/2}+\frac{b\left(\theta_{i-1/2}^{\left(\sigma \right),j+1}\right)}{h_{i}}r_{i-1/2}\right],\;F_{i}=\frac{1}{\tau_{j+1}}\theta_{i}^{j}+q_{lm}\frac{c\left(\theta_{i}^{j+1}\right)}{r_{i}}-\frac{\lambda_{pl}}{\tau }\frac{2a\left(\theta_{i}^{j+1}\right)}{r_{i}\left(h_{i}+h_{i+1}\right)}\left(\frac{b\left(\theta_{i+1/2}^{\left(\sigma \right),j+1}\right)}{h_{i+1}}r_{i+1/2}\left(\theta_{i+1}^{\left(\sigma +1\right),j}-\theta_{i}^{\left(\sigma +1\right),j}\right)\right.-\;\left.-\frac{b\left(\theta_{i-1/2}^{\left(\sigma \right),j+1}\right)}{h_{i}}r_{i-1/2}\left(\theta_{i}^{\left(\sigma +1\right),j}-\theta_{i-1}^{\left(\sigma +1\right),j}\right)\right).$$

The solution to the system of Equation (24) is sought in the following form

(25) $$y_{i}=\xi_{i+1}y_{i+1}+\zeta_{i+1},i=1,2,\ldots ,n-1,y_{n}=\zeta_{n+1},$$

where $\xi_{i+1}$ and $\zeta_{i+1}$ are the sweep coefficients.

The following recurrence relations are used to calculate the sweep coefficients:

$$\xi_{0}=\frac{B_{0}}{C_{0}},\xi_{i+1}=\frac{B_{i}}{C_{i}-A_{i}\xi_{i}},i=0,1,\ldots ,n-1,\;\zeta_{0}=\frac{F_{0}}{C_{0}},\zeta_{i+1}=\frac{F_{i}+A_{i}\zeta_{i}}{C_{i}-A_{i}\xi_{i}},i=0,1,\ldots ,n.$$

The initial values of the sweep coefficients $\xi_{0}$ and $\zeta_{0}$ are determined from the boundary conditions as follows:

$$\xi_{0}=1,\zeta_{0}=\frac{q_{lm}h_{1}-\frac{\lambda_{pl}}{\tau }g\left(\theta_{0}^{j}\right)\left(\theta_{1}^{j}-\theta_{0}^{j}\right)}{\left(1+\frac{\lambda_{pl}}{\tau }\right)g\left(\theta_{0}^{j}\right)}.$$

Because of this, we can calculate all the coefficients $\xi_{i}$, $\zeta_{i}$ up to i=n−1.

The definition of the quantity $h_{n}$ leads to the following quadratic equation:

(26) $$h_{n}^{2}+\tau_{j+1}q_{lm}h_{n}+\tau_{j+1}d\left(\theta_{i-1/2}^{j+1}\right)\left(\theta_{n}^{j+1}-\theta_{n-1}^{j+1}+\frac{\lambda_{pl}}{\tau }\left(\theta_{n}^{j+1}-\theta_{n-1}^{j+1}-\theta_{n}^{j}+\theta_{n-1}^{j}\right)\right)=0.$$

## 4. Numerical Results

All simulations were carried out under fully transient conditions. A fully implicit finite-difference scheme is employed. The nonlinear system (20) was solved at each time step using a simple iteration scheme, where convergence was evaluated using the residual $\underset{i}{\max}\left|\theta_{i}^{\left(\sigma +1\right),j+1}-\theta_{i}^{\left(\sigma \right),j+1}\right|$ with a tolerance of $\Delta =10^{-4}$. This iterative procedure typically required 3–5 iterations per time step. The initial spatial step was chosen in accordance with the characteristic length scale of the cake layer growth. As the moving boundary $R\left(t\right)$ advances, new grid nodes are introduced following the front-catching algorithm, with the boundary step $h_{n}$ recomputed at every time layer from Equation (26). The time step τ was chosen such that the condition $\tau <<\lambda_{pl}$ is satisfied, where $\lambda_{pl}$ is the pressure relaxation time. The relationship between the time step τ and the spatial boundary step $h_{n}$ is governed by Equation (26).

**Figure 3:**
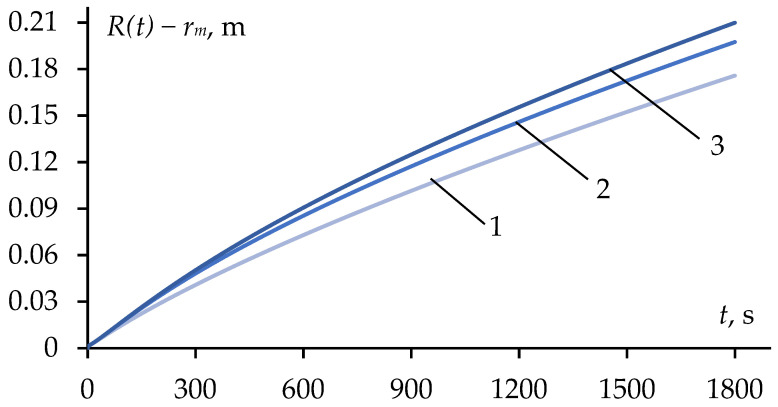
Dynamics of the cake layer thickness on the filter surface at $\lambda_{pl}=0$ (1); 150 (2); 300 (3) s.

**Figure 4:**
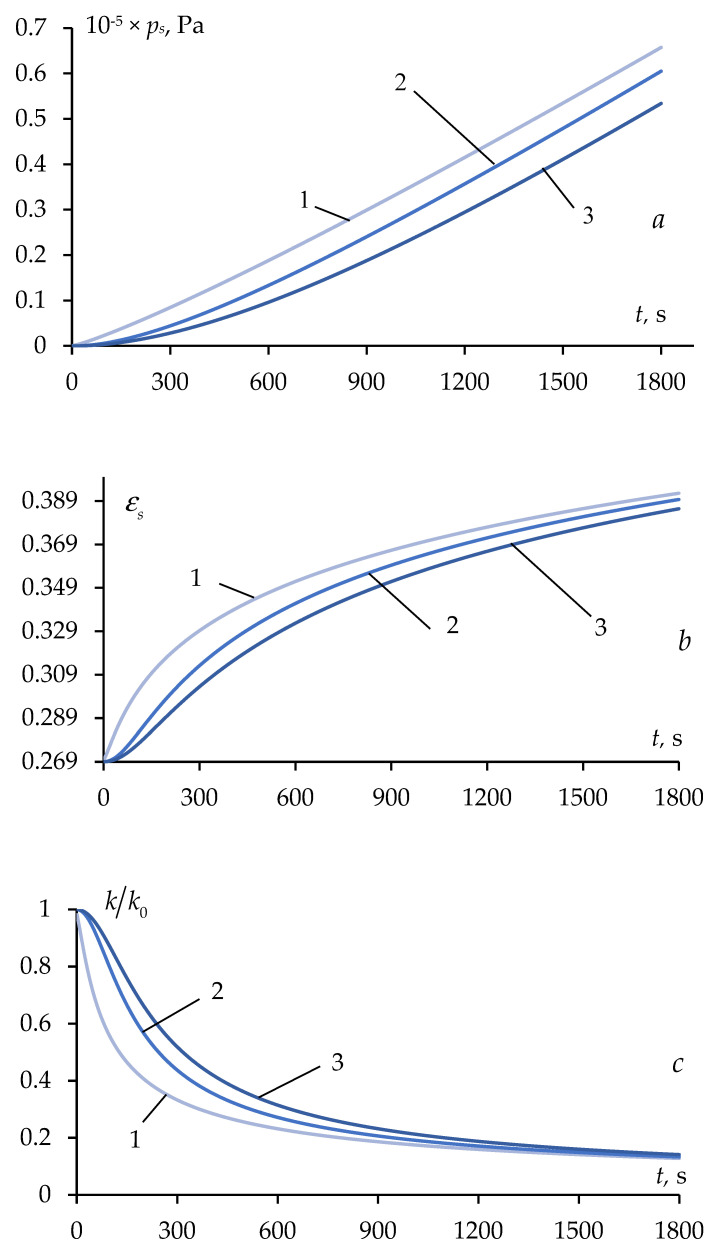
Dynamics of $p_{s}$ (**a**), $\varepsilon_{s}$ (**b**), $k/k_{0}$ (**c**) at point r=0.002 m at $\lambda_{pl}=0$ (1), $\lambda_{pl}=150$ (2), $\lambda_{pl}=300$ (3) s.

**Figure 5:**
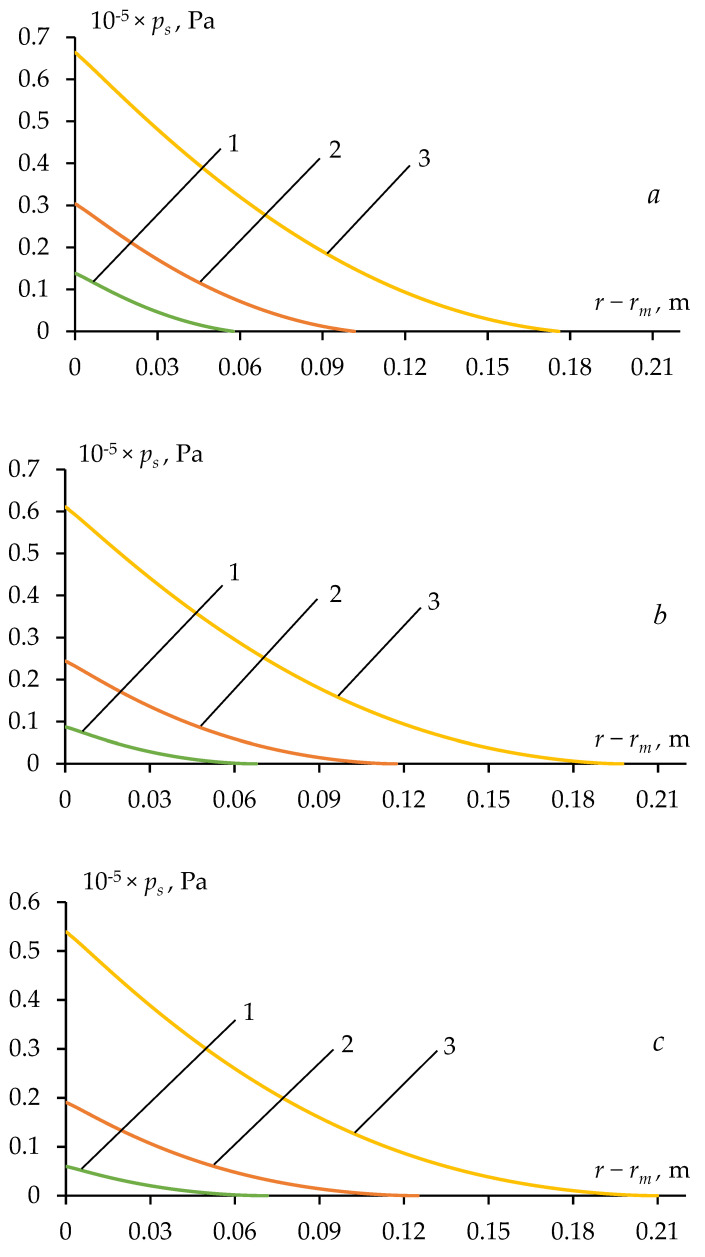
Distribution of compression pressure across the sediment thickness at t=450 (1); 900 (2); 1800 (3) s, $\lambda_{pl}=0$ (**a**), $\lambda_{pl}=150$ (**b**), $\lambda_{pl}=300$ (**c**) s.

**Figure 6:**
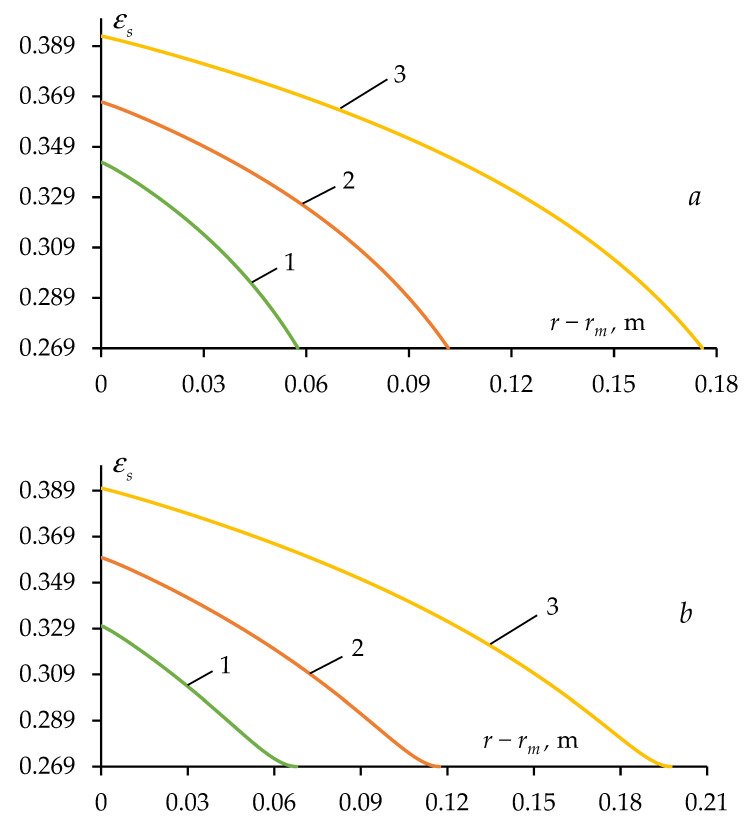

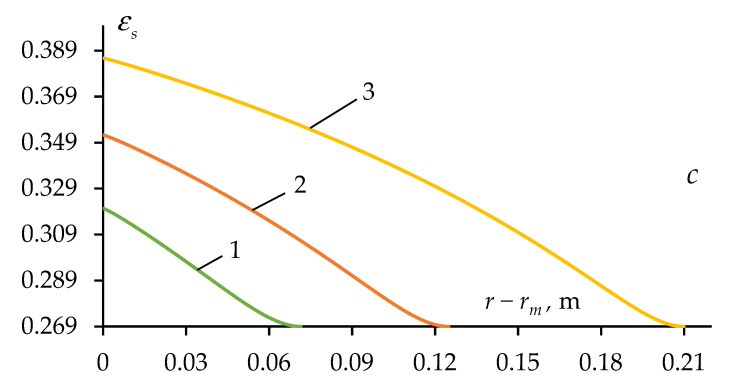
Change of $\varepsilon_{s}$ in sediment thickness at t=450 (1); 900 (2); 1800 (3) s, $\lambda_{pl}=0$ (**a**), $\lambda_{pl}=150$ (**b**), $\lambda_{pl}=300$ (**c**) s.

**Figure 7:**
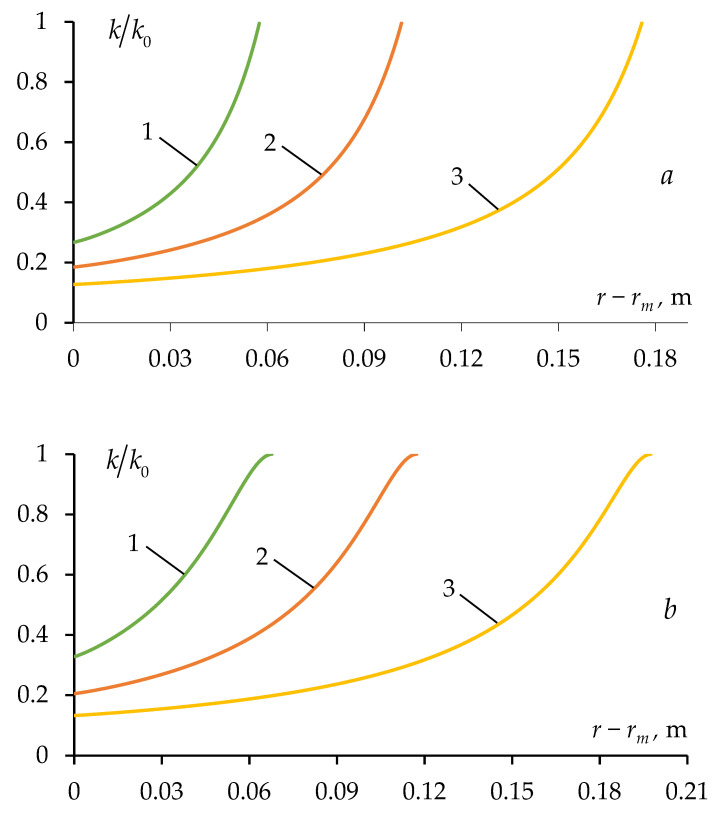

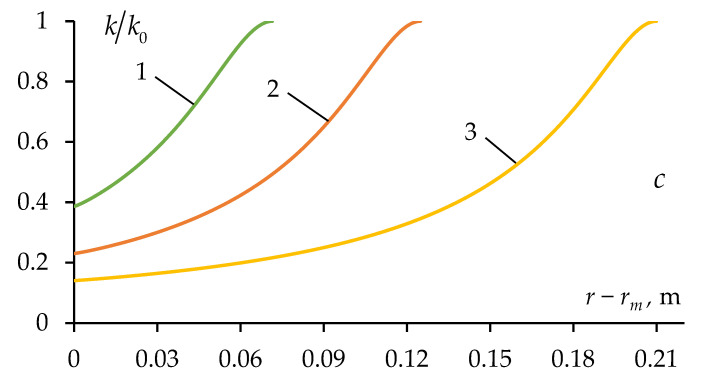
Change of $k/k_{0}$ in sediment thickness at t=450 (1); 900 (2); 1800 (3) s, $\lambda_{pl}=0$ (**a**), $\lambda_{pl}=150$ (**b**), $\lambda_{pl}=300$ (**c**) s.

Numerical results for (25) and (26) were obtained for the following values of the initial parameters [1]: $q_{lm}=2\cdot 10^{-5}$ m/s, $p_{A}=10^{3}$ Pa, $\mu =10^{-3}$ Pa·s, $k^{0}=3.4965\cdot 10^{-13}$ m^2^, $\varepsilon_{s}^{0}=0.269$, $\varepsilon_{s_{0}}=0.2$, β=0.09, δ=0.49. Some results are shown in Figure 3, Figure 4, Figure 5, Figure 6 and Figure 7.

Figure 3 presents graphical dependencies characterizing the growth of the cake layer thickness for different values of the relaxation time $\lambda_{pl}$. Analysis of these graphs shows that, all other things being equal, an increase in the relaxation time leads to a more intense growth of the cake thickness. This phenomenon indicates that the presence of relaxation effects in the mechanism of filtration flow under the influence of a pressure gradient significantly accelerates the process of formation of a layer of suspension particles on the filter surface. In particular, this is expressed in a change in the magnitude of the compression pressure and the degree of mechanical compaction of the solid phase.

Figure 4 shows the influence of the relaxation properties of the sediment on the dynamics of the main parameters of the filtration process at a fixed point r=0.002 m. Each curve corresponds to different values of the relaxation time $\lambda_{pl}$, with an increase in $\lambda_{pl}$ enhancing the manifestation of relaxation effects in the sediment. Figure 4a shows the change in compressive pressure over time. The nature of the curves shows that with an increase in the relaxation time $\lambda_{pl}$, the increase in compressive pressure slows down. This manifests itself as a “lagging” dynamics, which is due to the inertia of the deformation of the solid skeleton of the sediment: the higher the value of $\lambda_{pl}$, the slower the sediment particles adapt to changes in external pressure.

Figure 4b shows the change in porosity over time for different values of $\lambda_{pl}$. It is evident from the figure that the longer the relaxation time, the slower the decrease in porosity. For larger values of $\lambda_{pl}$, the porosity curve shifts upward relative to the non-relaxation case ($\lambda_{pl}=0$). This is explained by the “lagging” dynamics: the restructuring of the sediment and its compaction occur more slowly due to the presence of internal relaxation mechanisms. In particular, particles in the sediment require more time to reach a new equilibrium state. The relative permeability $k/k_{0}$ graphs (Figure 4c) characterize the dynamics of changes in filtration properties at a given point in the sediment depending on the relaxation time $\lambda_{pl}$. Analysis of the curves demonstrates that in the presence of relaxation effects, the decrease in permeability occurs with a certain delay relative to the equilibrium (non-relaxation) case. The longer the relaxation time $\lambda_{pl}$, the longer the sediment retains higher permeability values in the early stages of the process. This phenomenon is due to the inertia of the restructuring of the solid skeleton.

Figure 5, Figure 6 and Figure 7 show the profiles of compressive pressure, particle concentration, and sediment permeability for three time values and different relaxation times. With increasing time, the compressive pressure at the sediment (cake) boundary with the filter surface increases, indicating progressive compaction of the lower layers under the weight of new deposits and the effect of flow force (Figure 5). As the relaxation time $\lambda_{pl}$ increases, the peak pressure values decrease.

The highest solids concentration is observed at the filter membrane ($r-r_{m}$), where the cake is most compressed (Figure 6). Toward the outer sediment-suspension interface, the concentration drops to its initial value. High relaxation results in flatter parameter distribution profiles and a slower increase in the average cake density. This is explained by the fact that at relatively high values of $\lambda_{pl}$, the compaction process is distributed more evenly over time, preventing a sharp spike in concentration at the filter membrane in the initial stages of the process. This distribution profile confirms that intense particle consolidation occurs directly on the filter surface under the effect of maximum compressive pressure.

Minimum permeability is observed at $r-r_{m}$, where the particle concentration is maximum (Figure 7). At the sediment-suspension boundary, the ratio $k/k_{0}$ tends to 1, as this is where the newly deposited particles are located, not yet subjected to strong compression. Over time, the zone of low permeability (strong compaction) extends into increasingly deeper layers of the material. As the relaxation time $\lambda_{pl}$ increases, the permeability decrease slows, and the profiles $k/k_{0}$ become flatter. This is explained by the fact that at higher values of $\lambda_{pl}$, the particles adapt more slowly to increasing pressure, maintaining the sediment structure relatively loose and permeable for longer than in the equilibrium case.

**Figure 8:**
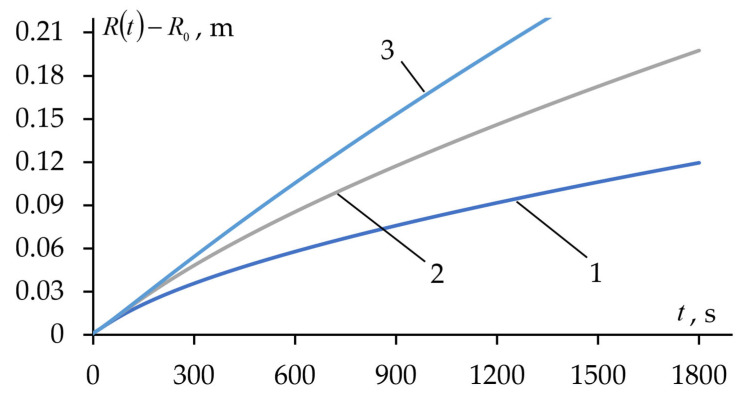
Dynamics of the sediment layer thickness on the filter surface at $\mu =10^{-2}$ (1); $10^{-3}$ (2); $10^{-4}$ (3) Pa·s, $\lambda_{p}=150$ s.

Figure 8 presents graphical dependencies characterizing the growth of the cake layer thickness for different values of the dynamic viscosity coefficient. Analysis of these graphs shows that, all other things being equal, an increase in fluid viscosity decreases the growth rate of the cake thickness. This is explained by the increase in hydrodynamic resistance within the porous medium as fluid viscosity increases, which consequently reduces the flow velocity. As the viscosity increases, the intensity of particle accumulation on the filter surface decreases due to the slowed-down flow, and the growth rate of the cake layer stabilizes.

**Figure 9:**
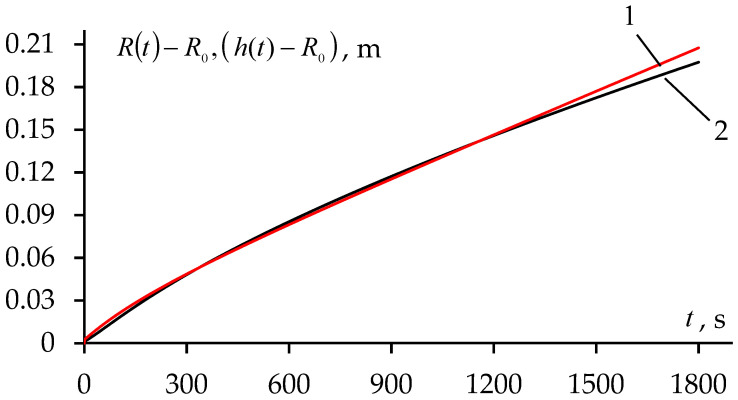
Dynamics of the cake layer thickness on the surface of flat (1) and radial (2) filters. $\lambda_{p}=150$ s.

Figure 9 presents a comparative analysis of the cake thickness dynamics on the surfaces of flat (1) and radial (2) filters under identical initial conditions and relaxation time. The analysis of the graphs indicates that during the initial stage of the filtration process, the growth rate of the cake layer thickness exhibits a nearly identical trend in both filter types. However, as time progresses, the cake thickness on the flat filter grows slightly more intensively compared to that on the radial filter.

## 5. Conclusions

This paper examines the relaxation filtration of a suspension through a radial filter surface at a constant flow velocity. Based on the adopted physical assumptions, a mathematical model of relaxation cake layer growth is formulated. This model includes continuity equations for the liquid phase, Darcy’s relaxation law, relationships for the compressive pressure and liquid phase pressure, and the dependence of the solid phase concentration and permeability on the compressive pressure. The fundamental equation of relaxation filtration, describing the pressure distribution in the cake, is represented as a nonlinear partial differential equation, where the desired function is the compressive pressure $p_{s}$. The Stefan condition, which determines the velocity of the moving cake layer boundary $R\left(t\right)$, is also formulated in terms of the compressive pressure gradient at the sediment-suspension interface. To numerically solve the problem with an unknown moving boundary, a version of the finite difference method with “front-catching” along the coordinate and the application of iterative methods is used.

The numerical results obtained demonstrate the significant role of relaxation effects in the dynamics of cake layer formation. It is shown that, over time, the compressive pressure and solids concentration increase, leading to a decrease in permeability and an increase in hydraulic resistance. Analysis of these graphs reveals that, all other things being equal, an increase in relaxation time leads to a more intense growth of cake thickness, as the slower compaction processes contribute to the maintenance of higher layer porosity over an extended period. Analysis of the pressure, concentration, and permeability distributions revealed that maximum cake compaction is observed near the filter surface. At the same time, pressure and solids concentration are at their lowest near the cake-slurry interface, while permeability is at its highest.

The obtained results allow us to identify the characteristics of relaxation filtration through cylindrical filters and can be used to optimize process parameters, evaluate filtration efficiency, and develop improved process control systems. The developed model and calculation methodology enable a reliable interpretation of the influence of the relaxation properties of the filtered system on the structure and growth dynamics of the cake layer.

## Data Availability

The original contributions presented in this study are included in the article. Further inquiries can be directed to the corresponding authors.
